# Astroglial S100B Secretion Is Mediated by Ca^2+^ Mobilization from Endoplasmic Reticulum: A Study Using Forskolin and DMSO as Secretagogues

**DOI:** 10.3390/ijms242316576

**Published:** 2023-11-21

**Authors:** Marina C. Leite, Fabiana Galland, Maria Cristina Guerra, Letícia Rodrigues, Jéssica Taday, Priscila T. Monteforte, Hanko Hirata, Carmem Gottfried, Rosario Donato, Soraya Smaili, Carlos-Alberto Gonçalves

**Affiliations:** 1 Departamento de Bioquímica, Universidade Federal do Rio Grande do Sul, Ramiro Barcelos, 2600-Anexo, Porto Alegre 90035-003, RS, Brazil; crisbareaguerra@hotmail.com (M.C.G.); letigues@gmail.com (L.R.); jessicataday@hotmail.com (J.T.); cgottfried@ufrgs.br (C.G.); casg@ufrgs.br (C.-A.G.); 2 Centro de Ciências e Qualidade dos Alimentos, Instituto de Tecnologia de Alimentos, Campinas 13070-178, SP, Brazil; fabianagalland@yahoo.com.br; 3 Departamento de Ciências Naturais, Universidade Federal de São João Del-Rei, São João Del Rei 36301-160, MG, Brazil; pris.farm@ufsj.edu.br; 4 Departamento de Farmacologia, Universidade Federal de São Paulo, São Paulo 04044-020, SP, Brazil; hanakoh@gmail.com (H.H.); soraya.smaili23@gmail.com (S.S.); 5 Interuniversity Institute of Myology, 06132 Perugia, Italy; rosario.donato47@gmail.com

**Keywords:** S100B secretion, calcium signaling, astrocytes

## Abstract

S100B, a homodimeric Ca^2+^-binding protein, is produced and secreted by astrocytes, and its extracellular levels have been used as a glial marker in brain damage and neurodegenerative and psychiatric diseases; however, its mechanism of secretion is elusive. We used primary astrocyte cultures and calcium measurements from real-time fluorescence microscopy to investigate the role of intracellular calcium in S100B secretion. In addition, the dimethyl sulfoxide (DMSO) effect on S100B was investigated in vitro and in vivo using Wistar rats. We found that DMSO, a widely used vehicle in biological assays, is a powerful S100B secretagogue, which caused a biphasic response of Ca^2+^ mobilization. Our data show that astroglial S100B secretion is triggered by the increase in intracellular Ca^2+^ and indicate that this increase is due to Ca^2+^ mobilization from the endoplasmic reticulum. Also, blocking plasma membrane Ca^2+^ channels involved in the Ca^2+^ replenishment of internal stores decreased S100B secretion. The DMSO-induced S100B secretion was confirmed in vivo and in ex vivo hippocampal slices. Our data support a nonclassic vesicular export of S100B modulated by Ca^2+^, and the results might contribute to understanding the mechanism underlying the astroglial release of S100B.

## 1. Introduction

S100B protein is a glial marker widely used in the investigation of brain damage and neurodegenerative and psychiatric diseases in patients and experimental models [1,2,3] in which its extracellular levels are evaluated in cerebrospinal fluid (CSF), blood serum, or cell culture medium. Changes in CSF and serum S100B are related to the inflammatory response in multiple sclerosis [4], and extracellular levels of S100B appear to have prognostic value for outcomes in traumatic brain injury [5].

S100B is a small (10.5 kDa) protein belonging to the S100 family of Ca^2+^-binding proteins [6], which comprises more than 20 members expressed in a cell-specific manner. Within cells, S100B exists as a homodimer in which the two subunits are held together by noncovalent bonds [7,8]. Astrocytes represent the major S100B-containing cell type in the gray matter of the central nervous system [1,9]. This protein has many putative intracellular targets but is also secreted (less than 1%) and has autocrine and paracrine effects on glia, neurons, and microglia. The effect of extracellular S100B on target cells can be trophic or toxic, depending on its local concentration, redox environment, and activated signaling pathways [7,10]. 

Many S100B secretagogues have been identified, including forskolin, lysophosphatidic acid [7], fluoxetine [11], kainate [12], huperzine A [13], and carbenoxolone [14]. Moreover, it is known that metabolic stress conditions affect S100B secretion, such as elevated concentrations of glutamate [15], glucose, beta-hydroxy-butyrate, and ammonia [1]. However, the underlying secretion mechanism remains unknown and possibly involves a nonclassical vesicular export [1,7,16].

S100B secretagogues have been studied in astrocyte cultures without fetal serum, used to grow and maintain cultured cells (e.g., [17]). However, serum deprivation per se is able to stimulate S100B secretion [7,18]. Therefore, some putative secretagogues might act via a mechanism triggered by serum deprivation, a complex event where Ca^2+^ might be a key mediator. For example, serum deprivation in astrocyte cultures induces Ca^2+^ release from the endoplasmic reticulum, which, in turn, causes the release of fibroblast growth factor-1 [19]. In addition, serum deprivation induces an early transient increase in cAMP [18], which could be associated with intracellular Ca^2+^ changes. However, a clear connection between intracellular Ca^2+^ in astrocytes and S100B release is still lacking.

We found that Ca^2+^-free medium or exposure to EGTA in acute hippocampal slices causes a significant increase in S100B secretion [20], in agreement with previous observations in brain slices [15], possibly due to the mobilization of internal stores of Ca^2+^. In fact, treatment with EGTA in a serum-free medium has been reported to cause an increase in S100B secretion from the U87 glioblastoma cell line [16]. In addition, Ca^2+^ channel blockers reduced S100B secretion in acute hippocampal slices [20], again suggesting an involvement of intracellular Ca^2+^. 

During the course of our study which aimed to investigate the role of extra- and intracellular Ca^2+^ in S100B secretion in primary astrocytes, we serendipitously observed that dimethyl sulfoxide (DMSO), a widely used vehicle in biological assays, is a powerful S100B secretagogue, and it is able to mobilizes intracellular Ca^2+^. Many other studies reported the biological activities of this compound (e.g., [21]). Herein, using different strategies to regulate intracellular Ca^2+^ (i.e., serum deprivation, forskolin, and DMSO), we characterized the mechanistic relation between S100B secretion and Ca^2+^ mobilization from the endoplasmic reticulum.

## 2. Results

### 2.1. Serum Deprivation, External Calcium, and Forskolin Stimulate S100B Secretion

**Figure 1:**
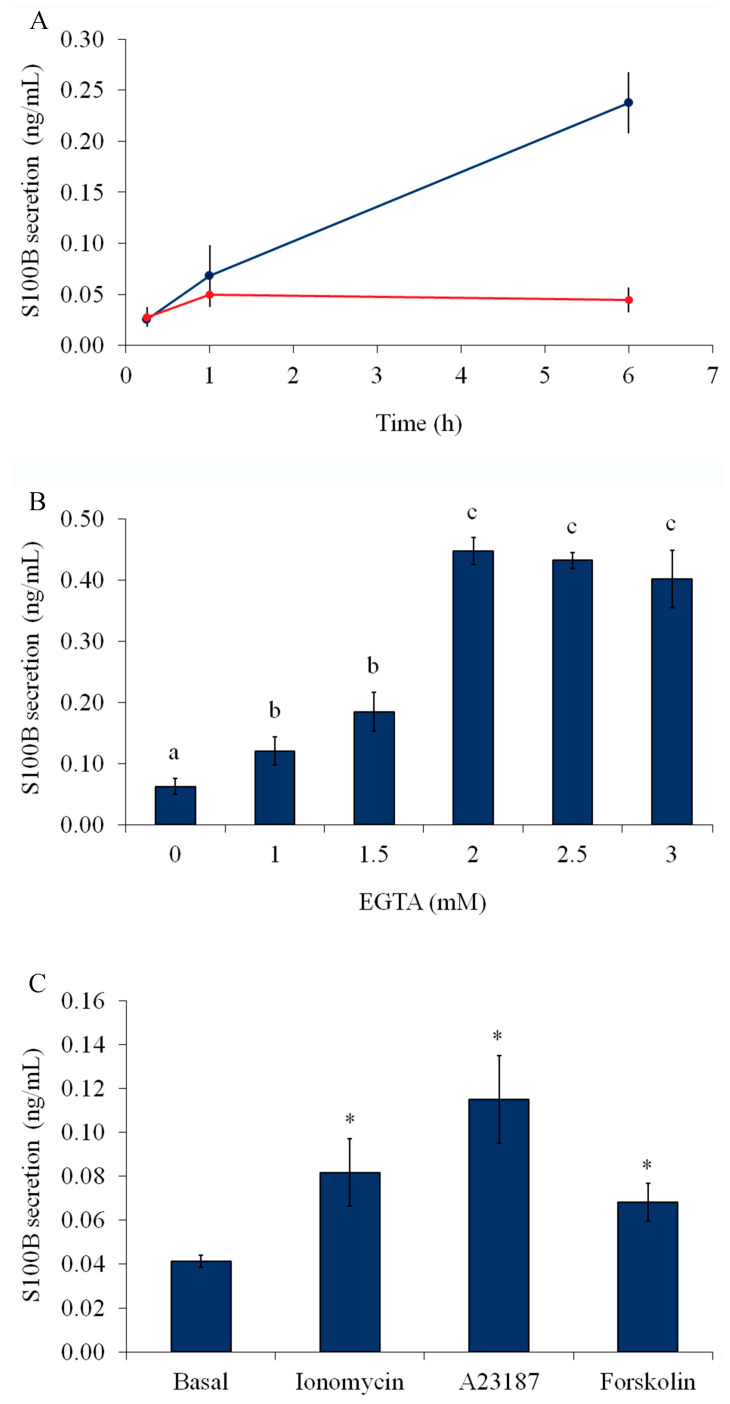
S100B secretion is stimulated by serum deprivation, removal of external calcium and forskolin. (**A**) Rat cortical astrocytes were cultured in DMEM containing 10% FCS. After confluence, the medium was replaced by the DMEM without serum (blue) or DMEM/10% FCS (red). S100B was measured by ELISA at 15 min, 1 h, and 6 h. Statistical analysis was carried out by repeated measures ANOVA. (**B**) The medium was replaced by DMEM without serum in the presence or absence of EGTA (from 1 to 3 mM). S100B was measured by ELISA at 1 h. Means indicated with a different letter differ (one-way ANOVA followed by Duncan’s test, assuming *p* < 0.05). (**C**) The medium was replaced by DMEM without serum in the presence or absence of 1 μM ionomycin, 1 μM A23187, or 10 μM forskolin. S100B was measured by ELISA at 1 h. * Significantly different from basal secretion (Student *t*-test followed by Bonferroni’s adjustment, *p* < 0.05). For all these results, each value represents the mean (±standard error) of at least 5 independent experiments performed in triplicate.

Generally, S100B secretion has been studied in glial cultures under serum-free conditions, in which Ca^2+^ and cAMP are putatively involved messengers [7,22,23]. A time-dependent increase in S100B secretion from astrocytes was observed after serum deprivation for 15 min, 1 h, and 6 h (Figure 1A). Figure 1B shows that EGTA (1–3 mM) significantly increased S100B secretion after 1 h of serum deprivation. This secretion was negatively correlated with external free Ca^2+^. In addition, ionophores (1 μM ionomycin or 1 μM A23187) were able to increase S100B secretion within 1 h; this increase was equivalent to or larger than that induced by forskolin, a well-known secretagogue for S100B (Figure 1C). Similar results were observed after 15 min for all these compounds (Figure S1). However, the secretion induced by ionophores, within 6 h, affected cell integrity (as evaluated by propidium iodide—Figure S2).

### 2.2. S100B Secretion Is Stimulated by DMSO, and This Effect Is Prevented by Thapsigargin or BAPTA-AM

**Figure 2:**
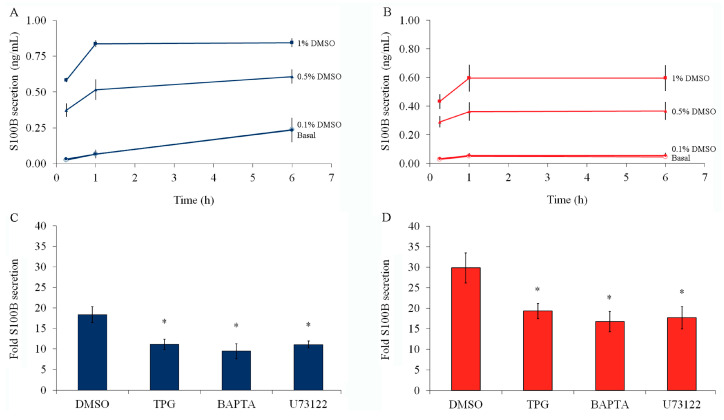
DMSO-induced S100B secretion, in the presence or absence of serum, was blocked by compounds that reduce intracellular mobilization of Ca^2+^. Rat cortical astrocytes were cultured in DMEM containing 10% FCS. After confluence, the medium was replaced by DMEM without serum ((**A**,**C**), blue) or DMEM/10% FCS ((**B**,**D**), red). In (**A**,**B**), DMSO was added at a final concentration ranging from 0.1 to 1%. S100B was measured by ELISA at 15 min, 1 h, and 6 h. Statistical analysis was carried out by repeated measures ANOVA. In (**C**,**D**), cells were treated with 1 μM thapsigargin (TPG), 10 μM BAPTA-AM, or 1 μM U73122 for 15 min before media replacement. Media were then replaced by DMEM without serum (in (**C**)) or DMEM/10% FCS (in (**D**)) containing 0.5% DMSO and the drug previously incubated. S100B was measured by ELISA after 1 h. Basal S100B secretion was assumed as 1 in each experiment. * Significantly different from DMSO-induced secretion (one-way ANOVA followed by Dunnett’s test, assuming *p* < 0.05). For all these results, each value represents the mean (±standard error) of at least 5 independent experiments performed in triplicate.

DMSO is the most common solvent for compounds used to mobilize or measure intracellular Ca^2+^. We investigated the effect of DMSO on S100B secretion. Interestingly, we found a positive correlation between DMSO concentration (up to 1%) and S100B secretion (Figure 2A) at all times examined (15 min, 1 h, and 6 h). Notably, ethanol (at 1%), another widely used solvent in biochemical assays, has no effects on S100B secretion (Figure S3). The dramatic DMSO-induced S100B secretion was also observed in the presence of serum (Figure 2B). 

DMSO-induced S100B secretion was significantly reduced by 1 µM thapsigargin (an inhibitor of the sarco/endoplasmic reticulum Ca^2+^-ATPase) or 10 µM BAPTA-AM (a permeable Ca^2+^ chelator which depletes intracellular Ca^2+^ levels), indicating that this effect is dependent on intracellular Ca^2+^ stores in the presence or absence of serum (Figure 2C and Figure 2D, respectively). Moreover, U73122 (at 1 μM), a phospholipase C (PLC) inhibitor, also reduced DMSO-stimulated S100B secretion. Notably, S100B secretion stimulated with serum deprivation was reduced by thapsigargin or BAPTA-AM (Figure S4).

### 2.3. Cell Viability and Integrity Are Not Affected by Forskolin or DMSO

**Figure 3:**
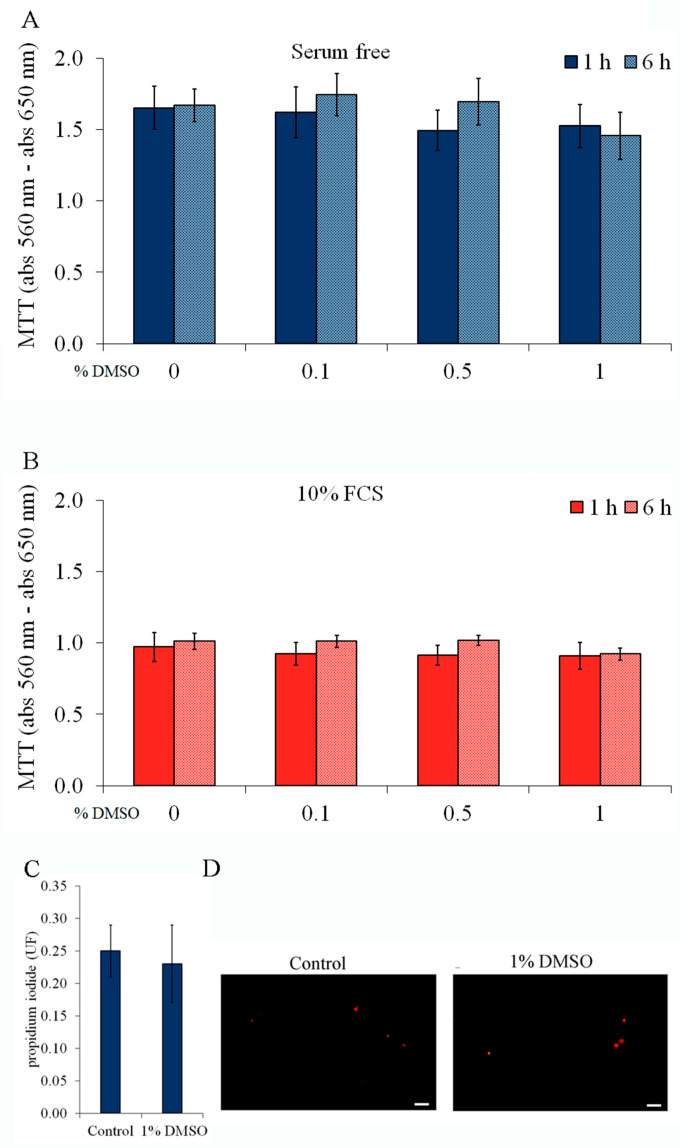
Cell integrity and cell viability were not affected by DMSO. Rat cortical astrocytes were cultured in DMEM containing 10% FCS. Confluent astrocytes were exposed to DMSO at a final concentration of 0.1, 0.5, or 1%, during 1 or 6 h. In (**A**,**B**), the MTT reduction assay was performed after 1 h (solid blue and red bars) or 6 h (dotted blue and red bars) of incubation in DMEM without serum (in (**A**), blue) or with 10% FCS (in (**B**), red). Statistical analysis was performed by one-way ANOVA. In (**C**,**D**), astrocytes were incubated with 7.5 μM propidium iodide (**C**) iodide propidium fluorescence quantification after 6 h of incubation in DMEM with 10% FCS, in the absence or presence of 1% DMSO. Statistical analysis was performed by Student *t*-test. (**D**) representative propidium iodide fluorescent images of astrocytes after 6 h of incubation in DMEM with 10% FCS, in the absence (at left) or presence of 1% DMSO (at right). Scale bar: 50 µm. For all these results, each value is the mean (±standard error) of at least 4 independent experiments performed in triplicate.

Two assays were performed to characterize cell viability: the MTT reduction assay (Figure 3A,B) and PI exclusion (Figure 3C). DMSO (0.1–1%) did not affect the cell capacity of MTT reduction at 1 or 6 h either in the absence (Figure 3A) or in the presence (Figure 3B) of serum. Notably, the MTT reduction was significantly higher (~60%) in serum-deprived astrocytes (Student *t*-test, *p* < 0.05, in basal conditions or in the presence of DMSO). DMSO (at 1%) did not cause changes in cell integrity, based on PI exclusion assay, (Figure 3C,D) of serum at 6 h. Similar results were obtained with forskolin (Figure S5). Thus, DMSO-induced S100B secretion was not due to cell viability and/or integrity changes. Taken together, these data rule out a nonspecific effect of DMSO on membrane permeability or alterations in cell viability that could explain S100B efflux.

### 2.4. Forskolin, EGTA, and DMSO Induce an Increase in Intracellular Ca^2+^

**Figure 4:**
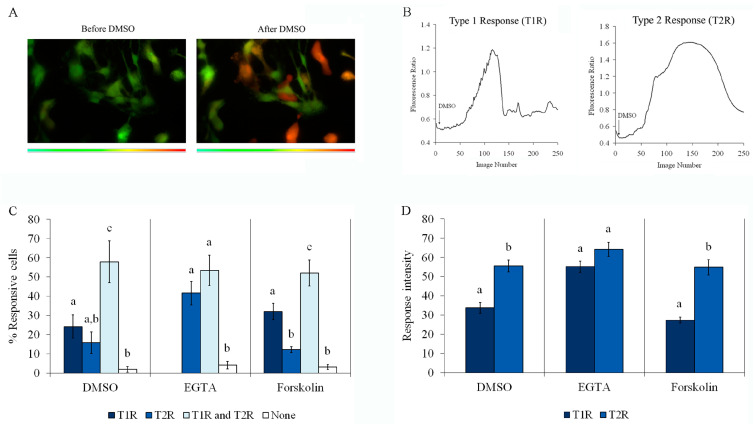
Forskolin, EGTA, and DMSO induce an increase in intracellular Ca^2+^ in astrocytes. Rat cortical astrocytes were cultured in DMEM containing 10% FCS. Intracellular calcium variations were evaluated in preconfluent astrocytes loaded with Fura-2 by real-time fluorescence microscopy. (**A**) Representative fluorescent images of cells loaded with Fura-2 before and after DMSO stimulation (20× magnification). (**B**) Representative graphs of the type 1 or type 2 responses (T1R and T2R, respectively) induced by DMSO. (**C**) Percentage of responsive and nonresponsive cells exposed to 0.5% DMSO, 2 mM EGTA, or 10 µM forskolin. Cells that exhibited only type 1 response (T1R) are represented by dark blue bars, cells that exhibited only type 2 response (T2R) are represented by blue bars, cells that exhibited T1R and T2R responses are represented by light blue bars, and nonresponsive cells are represented by white bars. Means, in each stimulating condition, indicated by different letters differ (one-way ANOVA followed by Duncan’s test). (**D**) Intensity of cell response to 0.5% DMSO, 2 mM EGTA, or 10 µM forskolin, compared with response induced by 0.1 mM digitonin (assumed as 100%): intensities of T1R and T2R are represented by dark blue bars and blue bars, respectively. Means, in each stimulating condition, indicated by different letters differ (Student *t*-test, assuming *p* < 0.05).

To investigate whether DMSO-stimulated S100B secretion was dependent on cytosolic Ca^2+^ concentration, we first analyzed the intracellular Ca^2+^ response, as measured by Fura-2 fluorescence, in primary astrocytes exposed to DMSO compared to cells treated with forskolin or EGTA. Representative images of astrocytes loaded with Fura-2 before and after DMSO are shown in Figure 4A. Two types of Ca^2+^ response were induced by DMSO, as shown by graphs in Figure 4B, where a fast, short, and transient increase (type 1 response, T1R) and a slower and more sustained increase (type 2 response, T2R) can be observed. It is important to emphasize that cells from each field were individually analyzed and showed a heterogeneous calcium response profile. While some cells responded by exhibiting only T1R or T2R, others showed a biphasic response (T1R and T2R, during the period of analysis) and others did not respond at all. Quantitative analysis of assays with Fura-2 were performed to calculate the percentage of cells responsive to 0.5% DMSO or 2 mM EGTA (resulting in a Ca^2+^-free medium) or 10 µM forskolin (Figure 4C), and the intensity of these responses compared to the maximum fluorescence obtained with digitonin, which was added at the end of the period of incubation (Figure 4D). Figure 4C shows the percentage of cells that responded with T1R only, T2R only, and both T1R and T2R, and cells with no response (none) after DMSO, EGTA, or forskolin addition. Note that DMSO and forskolin induced T1R only in 25 and 30% of cells, respectively, while T1R only was not observed during incubation with EGTA. Moreover, less than 20% of cells exhibited TR2 only following treatment with DMSO or forskolin, while more than 40% exhibited T2R only during incubation with EGTA. Under all conditions, the percentage of cells exhibiting T1R and T2R was higher than 50%, and less than 5% were nonresponsive (neither T1R nor T2R). Thus, the profile of the DMSO-induced Ca^2+^ increase was more similar to that evoked by forskolin than that caused by the addition of EGTA. Further, the intensities of T1R and T2R (Figure 4D) during incubation with EGTA were not different, but they differed (with T2R > T1R) when they were induced by DMSO or forskolin. 

### 2.5. DMSO-Induced Ca^2+^ Increase Involves Mobilization from the Endoplasmic Reticulum

**Figure 5:**
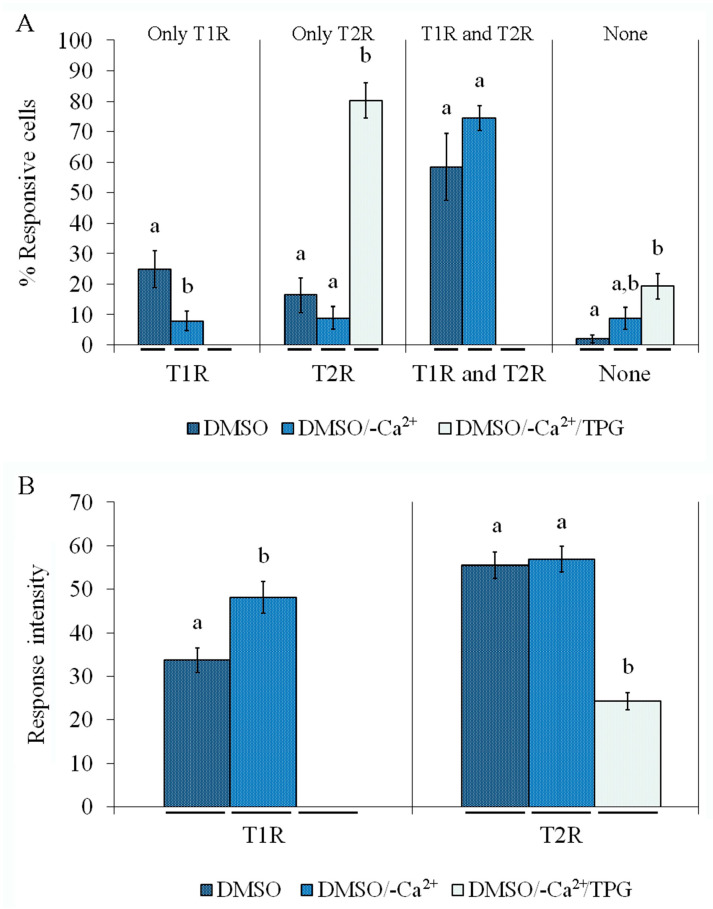
DMSO-induced intracellular calcium mobilization affected by thapsigargin and external calcium. Intracellular calcium variations were evaluated in preconfluent astrocytes loaded with Fura-2 by real-time fluorescence microscopy, stimulated by 0.5% DMSO (dotted dark blue bars) or DMSO in a calcium-free medium (DMSO/-Ca^2+^, dotted blue bars) or DMSO in a calcium-free medium containing 1 µM thapsigargin (DMSO/-Ca^2+^/TPG, dotted light blue bars). (**A**) Percentage of cells exhibiting only a type 1 response (T1R); only a type 2 response (T2R); both T1R and T2R; and neither T1R nor T2R (none) were analyzed separately, comparing the different stimulating conditions. (**B**) Intensities of type 1 and type 2 responses (T1R and T2R, respectively) obtained under different stimulating conditions. Means, under each stimulating condition, indicated by a different letter differ (one-way ANOVA followed by Duncan’s test, assuming *p* < 0.05).

In another set of experiments, we investigated the involvement of Ca^2+^ from the external medium (by removing it with EGTA) and/or from the endoplasmic reticulum (by adding thapsigargin) in the types of responses: T1R; T2R; TR1 and T2R; and neither T1R nor T2R (none) (Figure 5A). About 25% of cells exhibited T1R in the presence of DMSO, which fell to 10% in the absence of external Ca^2+^; however, this response disappeared when cells were incubated with thapsigargin in a Ca^2+^-free medium. On the other hand, the percentage of cells that exhibited T2R in the presence of DMSO was not altered by the removal of external Ca^2+^ and dramatically increased when cells were incubated in the presence of EGTA plus thapsigargin. The percentage of cells that exhibited T1R and T2R under the influence of DMSO (about 60%) was not altered by the removal of external Ca^2+^ as well but disappeared when thapsigargin was added. It was also possible to observe an increase in the percentage of nonresponsive cells in EGTA-treated cultures and even more so in EGTA/thapsigargin-treated cultures (graph on the right in Figure 5A). In addition, the intensity of T1R (Figure 5B) induced by DMSO was higher in a Ca^2+^-free medium, and interestingly, it was abolished entirely when thapsigargin was added. The removal of Ca^2+^ did not modify the intensity of DMSO-induced T2R but significantly decreased when thapsigargin was added to the Ca^2+^-free medium. Collectively, these results suggested that (1) DMSO induces cytosolic Ca^2+^ increases via mobilization from the endoplasmic reticulum, and (2) DMSO-induced S100B secretion might be dependent on cytosolic Ca^2+^ increases. In other words, whereas T1R was abolished by this treatment, the T2R (i.e., sustained) response was not completely inhibited by Ca^2+^-free medium plus thapsigargin, indicating that DMSO may mobilize Ca^2+^ from intracellular stores not sensitive to thapsigargin. This source of Ca^2+^ might be cell compartments other than the endoplasmic reticulum, such as the mitochondria and/or lysosomes, which may participate in Ca^2+^ signaling and homeostasis in astrocytes [24,25,26].

### 2.6. Inhibition of Plasma Membrane Ca^2+^ Channels Reduces S100B Secretion

**Figure 6:**
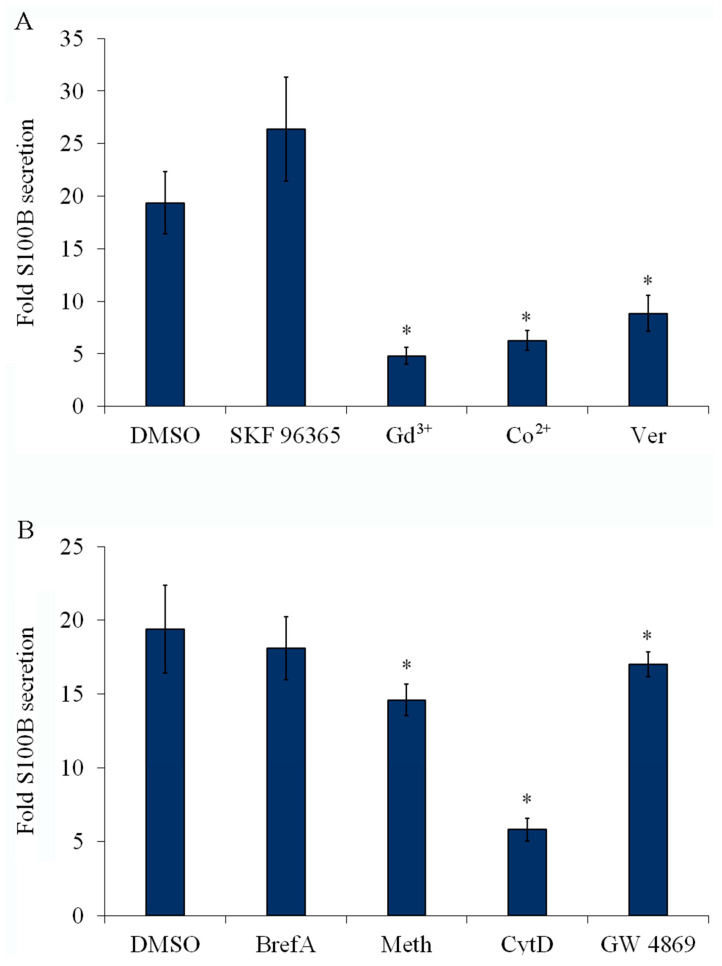
DMSO-induced S100B secretion affected by Ca^2+^ channel blockers of plasma membrane, methylamine, and cytochalasin D. Rat cortical astrocytes were cultured in DMEM containing 10% FCS. After confluence, the medium was replaced by the DMEM without serum, and DMSO was added at a final concentration of 0.5%. In (**A**), fold S100B secretion induced by DMSO (assuming basal S100B secretion as 1 in each experiment) in the presence of channel blockers: 0.2 mM SK F96365, 0.5 mM, GdCl_3_ (Gd^3+^), 1 mM CoCl_2_ (Co^2+^), 50 μM verapamil (Ver). In (**B**), S100B secretion in the presence of 5 μM brefeldin A (BrfA), 10 mM methylamine (Meth), 10 μM cytochalasin D (CytD), or 10 μM GW4869. Each value is the mean (± standard error) of at least 5 independent experiments performed in triplicate. * Significantly different from DMSO-induced secretion (one-way ANOVA followed by Dunnett’s test, assuming *p* < 0.05).

Next, we sought to determine the role of the replenishment of intracellular Ca^2+^ stores in S100B secretion using a pharmacological approach. SKF 96365, an inhibitor of store-operated Ca^2+^-entry (SOCE), was unable to block S100B secretion induced by DMSO (Figure 6A). In addition, incubation with an anti-TRPC1 antibody alone also was unable to reduce S100B secretion (Figure S6). However, gadolinium (Gd^3+^) and cobalt (Co^2+^), two cations widely used for blocking SOCE and Ca^2+^ channels in general, respectively, decreased DMSO-induced S100B secretion (Figure 6A). Verapamil, an inhibitor of voltage-gated L-type Ca^2+^ channel, also inhibited DMSO-induced S100B secretion (Figure 6A). Similar results were obtained when S100B secretion was stimulated with forskolin (Figure S7). These results suggested that DMSO- and forskolin-induced S100B secretion requires the activity of plasma membrane Ca^2+^ channels.

### 2.7. S100B Secretion Involves a Vesicle-Exporting Mechanism

S100B-containing vesicles have been reported in the culture medium of glioma cells (e.g., [16]). Thus, we investigated some basic steps that could be associated with the vesicular export of proteins driven by Ca^2+^ mobilization. To this end, DMSO-treated astrocytes were incubated with either brefeldin A (a classical inhibitor of protein traffic via the Golgi), methylamine (an inhibitor of vesicle acidification), or cytochalasin D (an F-actin disassembling agent). Confirming our expectation, brefeldin A did not affect S100B secretion, whereas both methylamine and cytochalasin D reduced S100B secretion (Figure 6B). Lastly, based on the presence of S100B in serum S100B-containing exosomes [27], we investigated S100B secretion using GW4869, an inhibitor of the neutral sphingomyelinase 2, involved in the formation of astrocyte exosomal vesicles [28]. In fact, in the presence of this inhibitor, we observed that DMSO-stimulated S100B secretion dropped by approximately 15%.

### 2.8. Different from Forskolin, DMSO Does Not Induce Morphological Changes Associated with S100B Secretion

**Figure 7:**
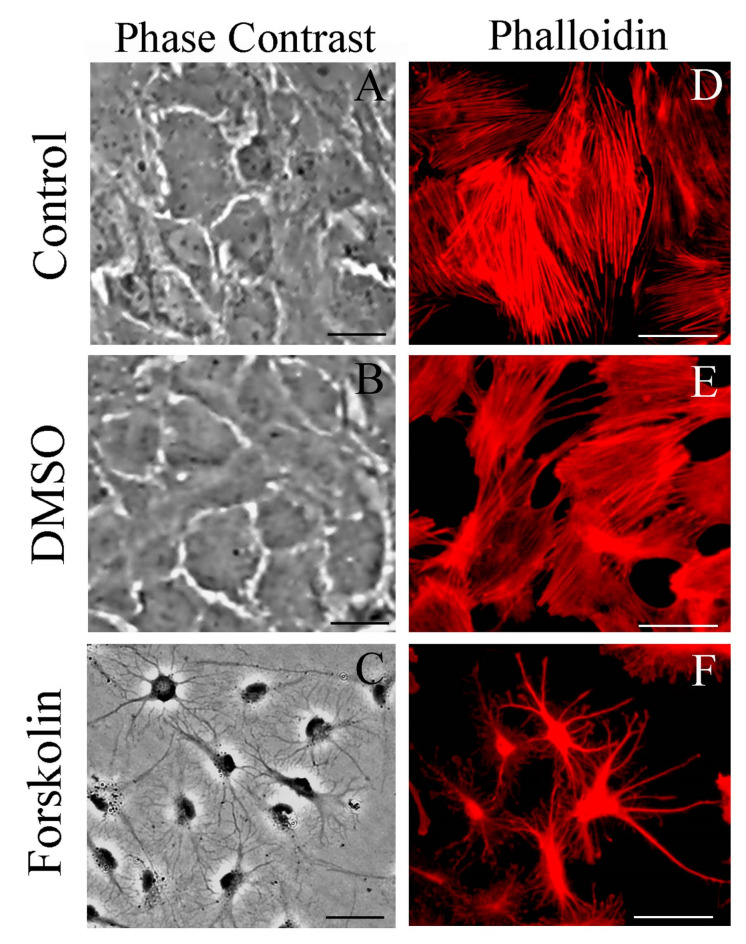
Forskolin, but not DMSO, induced morphological changes associated with S100B secretion. Rat cortical astrocytes were cultured in DMEM containing 10% FCS. Confluent and preconfluent cells in basal conditions (**A**) or incubated with 1% DMSO (**B**,**E**) or incubated with 10 μM forskolin for 1 h (**C**,**F**) were analyzed by phase-contrast microscopy (**A**–**C**) and fluorescent microscopy after rhodamine-phalloidin staining for actin filaments (**D**–**F**). Images are representative of 4 independent experiments. Scale bar: 50 µm.

Phase-contrast microscopic images indicate that serum deprivation does not cause a significant stellation in primary astrocytes at 1 h after treatment. Cytochemical staining with phalloidin for actin confirmed typical flat and polygonal cells (Figure 7, panels A and D, respectively). The addition of DMSO (at 1%) also did not alter this morphology (panels B and E); however, forskolin (at 10 mM) induced a high degree of stellation (panels C and F).

### 2.9. The Effect of DMSO on S100B Secretion Was Also Observed In Vivo and in Acute Hippocampal Slices

**Figure 8:**
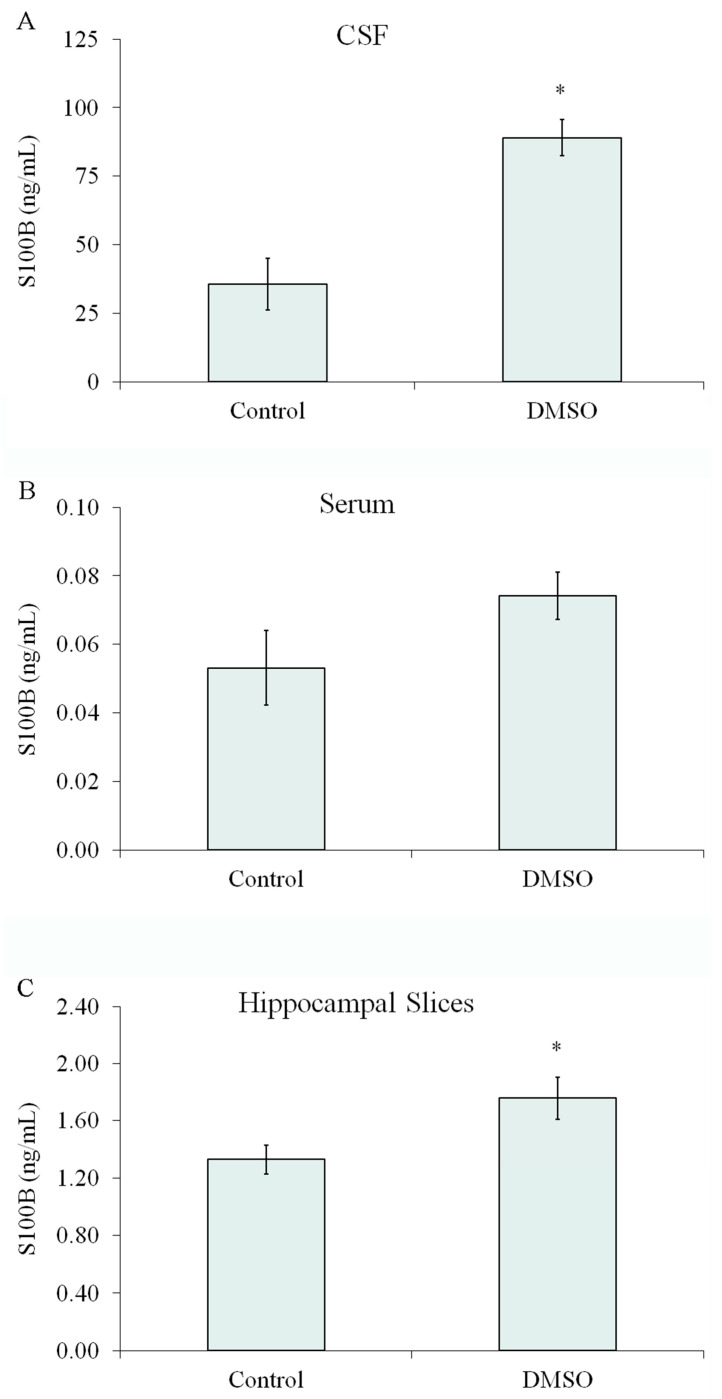
DMSO increased cerebrospinal content of S100B and in vitro S100B secretion in brain slices. (**A**,**B**) Intracerebroventricular injection of DMSO (2 μL) or saline solution (2 μL) in adult Wistar rats under anaesthesia. After 1 h, cerebrospinal fluid and blood were collected by magna puncture and intracardic puncture, respectively. Cerebrospinal fluid (**A**) and serum (**B**) contents of S100B were measured by ELISA. Each value represents the mean (±standard error) of 5 rats per group. (**C**) Adult Wistar rats were killed by decapitation, and 0.3 mm hippocampal slices were obtained using a McIlwain chopper. After a metabolic recovery period, hippocampal slices were exposed to 1% DMSO, and extracellular content of S100B was measured by ELISA. Each value is the mean (±standard error) of at least 4 independent experiments performed in triplicate. * Significantly different from the respective control (Student *t*-test, *p* < 0.05).

Anesthetized rats received 2 µL intracerebroventricular (ICV) of DMSO or phosphate-buffered saline (control). Assuming a CSF volume of 200 µL (excluding subarachnoid space) [29], the final DMSO concentration was calculated to be approximately 1%. CSF was collected by cisterna magna puncture at 1 h after, and a significant increase in S100B was observed at this time point (Figure 8A), without significant changes in S100B serum content (Figure 8B). Moreover, a significant increase in S100B secretion was found in ex vivo hippocampal slices when exposed to 1% DMSO (Figure 8C).

## 3. Discussion

**Figure 9:**
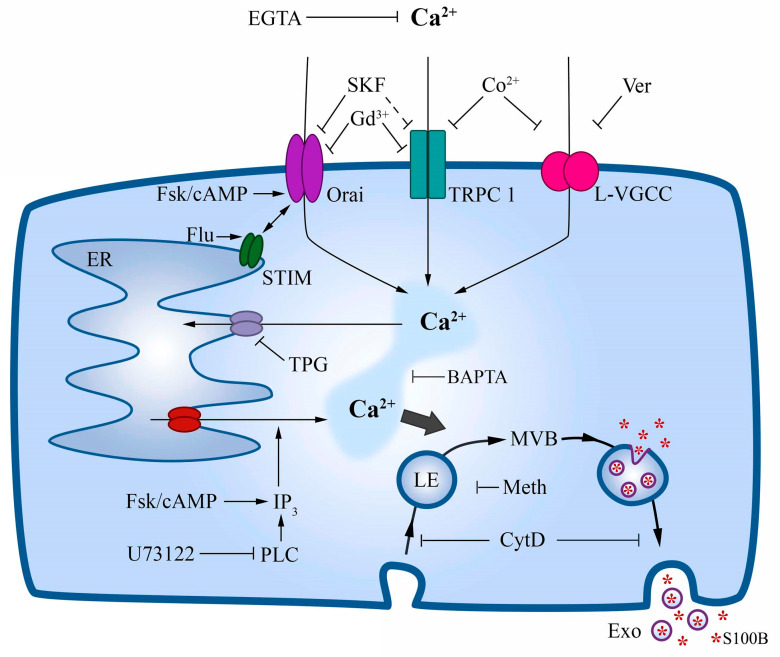
Schematic representation of the S100B secretion modulated by intracellular Ca^2+^. Ca^2+^ is mobilized from endoplasmic reticulum (ER), leading to S100B secretion by exocytosis. Forskolin via cyclic AMP stimulates replenishment and release from Ca^2+^ ER store. DMSO, like forskolin, stimulates Ca^2+^ release from RE and S100B secretion, but molecular target(s) is (are) unknown. Flufenemic acid (Flu) stimulates STIM and S100B secretion. All pharmacological inhibitors used in S100B release assays reduced Ca^2+^ flow and S100B secretion, except EGTA, which by external Ca^2+^ removal stimulates internal Ca^2+^ mobilization. Two Ca^2+^ ionophores (see Figure 1) also support this view. Plasma membrane Ca^2+^ channels (e.g., TRPC1 and L-VGCC) are involved in the replenishment of internal stores. S100B exocytosis regulated by Ca^2+^ was reduced by methylamine and cytochalasin D. Abbreviations: CytD, cytochalasin D; ER, endoplasmic reticulum; Exo, exosomes; Flu, flufenamic acid; Fsk, forskolin; IP3, inositol tris-phosphate; L-VGCC, L-type voltage-gated calcium channel; LE, late endosome; Meth, methylamine; MVB, multivesicular body; PLC, phospholipase C; SKF, SKF 96365, a store-operated Ca^2+^ entry inhibitor that inhibits STIM1; STIM, stromal interaction molecule; TPG, thapsigargin; TRPC1, transient receptor potential canonical channel, type 1; Ver, verapamil. S100B is represented by a red asterisk.

Astrocytes express and secrete S100B protein, and extracellular levels of this protein have been used as a marker of brain injury and glial activation (e.g., [30]). There is abundant in vitro evidence that extracellular S100B plays a trophic or toxic role in neural cells at low and high concentrations, respectively [7,31]. However, the mechanism of S100B secretion remained unclear. A nonclassical route for S100B release has been proposed in which Ca^2+^ might be a mediator [16]. Also, in most cell culture assays, basal S100B secretion is enhanced by serum deprivation (e.g., [7,32]), which per se mobilizes intracellular Ca^2+^ in astrocytes [19]. Moreover, the removal of external Ca^2+^ in nonexcitable cells, like astrocytes, induces Ca^2+^ mobilization from internal Ca^2+^ stores and S100B secretion [14,16,33,34]. These results indicate a relationship between increases in cytosolic Ca^2+^ concentration and S100B secretion; however, a mechanistic link between these two events was missing. Herein, using primary cortical astrocytes, we show that S100B secretion negatively correlates with the external concentration of Ca^2+^ as S100B secretion increases proportionally to decreased external Ca^2+^, and this effect depends on time. Further, we show that Ca^2+^-ionophores induce S100B secretion, and factors interfering with cytosolic Ca^2+^ increases also reduce S100B release. Thus, increases in cytosolic Ca^2+^ levels appear to lead to S100B secretion, irrespective of the mechanism of cytosolic Ca^2+^ increase, as schematically represented in Figure 9.

DMSO is an amphipathic molecule with a highly polar sulfoxide [-S-O] and two apolar dimethyl groups [-(CH_3_)_2_], making it a very good vehicle in both aqueous and organic media. This compound is often assumed to be a safe and inert vehicle or is used at safe concentrations for specific assays. The concentration of DMSO used as a vehicle varies from low levels, such as 0.1%, to higher levels, such as 20%. However, some effects (and side effects) of this agent are neglected. Due to its properties, particularly as a radical scavenger and hydrogen-bond disrupter, this membrane-diffusible solvent can affect both in vitro and in vivo assays [35]. With regard to protein secretion, DMSO has been reported to increase LPS-induced secretion of interleukin-1 in human monocytes [36], augment glucagon-like peptide1-induced secretion of insulin in a pancreatic β-cell line [37], and decrease homocysteine-induced secretion of interleukin-8 in human monocytes [38]. 

We found that DMSO increased S100B secretion in a concentration- and time-dependent manner. The S100B secretion was robust compared to a well-known secretagogue, such as forskolin [7], irrespective of the absence or presence of serum. An effect of DMSO on S100B secretion involving Ca^2+^ release from internal stores is supported by the inhibitory effect exerted by BAPTA-AM and thapsigargin (dissolved in DMSO at 0.04%). In addition, significant inhibition of S100B secretion was obtained with U73122, an inhibitor of PLC, suggesting that IP_3_ might be involved.

DMSO (at 0.5% or 1%) induced an increase in intracellular Ca^2+^, and the profile of this effect (shape and amplitude of the responses) was more similar to that obtained with forskolin than that obtained by removal of external Ca^2+^ with EGTA. The secretagogue effect of forskolin on S100B could be explained by a stimulation of IP_3_-sensitive Ca^2+^ channels by PKA [39]. Accordingly, H89, an inhibitor of PKA, reduced the S100B secretion induced by serum deprivation [18], and herein, thapsigargin, an inhibitor of the endoplasmic reticulum Ca^2+^-ATPase, reduced the S100B secretion induced by serum deprivation and forskolin. Astrocytes express type 2 IP_3_ channels [40], which apparently (at least in non-neural cells) are weakly sensitive to PKA phosphorylation [41]. However, cAMP/PKA appears to stimulate Ca^2+^ release from IP_3_-sensitive stores for glutamate exocytosis in astrocytes [42]. An alternative explanation might be that the cAMP/PKA system induces cytoskeleton reorganization, which in turn remodels the space between the plasma membrane and the endoplasmic reticulum, increasing Ca^2+^ flow into the cells [43]. Moreover, forskolin/cAMP stimulates the Orai channels of store-operated Ca^2+^ entry (SOCE) [44,45]. These possibilities are indicated in Figure 9. DMSO induced a forskolin-like profile of intracellular Ca^2+^ increase, possibly also mobilizing Ca^2+^ from internal stores. However, an indication of any molecular target(s) is merely speculative at present.

In astrocytes, Ca^2+^-entry occurs via Ca^2+^ channels regulated by chemical signals (including neurotransmitters) and voltage, whereas the replenishment of internal stores by SOCE is carried out by a complex mechanism involving the Ca^2+^-sensor STIM (stromal interaction molecule) protein and the Ca^2+^-channels Orai and TRPC [46,47] that operate synergistically [48]. Thus, we investigated the effect of pharmacological inhibitors of Ca^2+^ channels on S100B secretion. SKF 96365, a direct store-operated Orai channel antagonist, was unable to reduce DMSO-induced S100B secretion. It is important to mention that in glioblastoma cells, SKF 96365 increases the reverse operation of the Na^+^/Ca^2+^ exchanger, causing an increase in intracellular Ca^2+^ [49]. Moreover, although this inhibitor is used as a general SOCE inhibitor, it is unclear if it targets TRPC1, the main TRPC channel involved in Ca^2+^ replenishment in astrocytes [46]. Contrariwise, Gd^3+^, which blocks Orai and TRPC channels, was able to reduce S100B secretion. Cobalt (Co^2+^), a general blocker of Ca^2+^ channels, inhibited S100B secretion. Moreover, verapamil, an inhibitor of voltage-gated L-type Ca^2+^ channel, also inhibited DMSO-induced S100B secretion. These results reinforce previous results in acute hippocampal slices, where Co^2+^ and verapamil inhibited S100B secretion [20]. 

S100B does not exhibit the specific sequences typical of proteins secreted in the classical vesicular way, i.e., via the Golgi system. Herein, as in previous works, brefeldin A did not affect S100B secretion at 1 h or 6 h either with forskolin or DMSO stimulation. However, large S100B-containing vesicles were described in U373 and S100B-transfected U87 glioblastoma [16]. In light of current knowledge, those large vesicles could correspond to ectosomes, more commonly found in tumor cells [50]. To our knowledge, no large S100B-containing vesicles have been described in astrocytes, and we failed to find such vesicles by confocal microscopy, a methodologically complex procedure considering the huge amount of this protein in the cytosol. More recently, endocytic vesicles in Schwann cells [51] and astrocytes [52] containing Alexa-labelled S100B have been reported. Such endocytic uptake of S100B could support the exocytic way of S100B secretion. When we incubated astrocytes with methylamine, an endocytosis inhibitor [53], S100B secretion was reduced, suggesting that S100B secretion depends on vesicle acidification. Therefore, it is possible to conceive an exocytic way for S100B secretion in astrocytes via exosomes, formed from a multivesicular body, where part of cytosolic S100B is carried within intraluminal vesicles. In support of this possibility, S100B-containing exosomes were described in serum [27]. Cytochalasin D, an F-actin-disrupting agent, reduced the S100B secretion induced by DMSO, suggesting a vesicular movement dependent on the F-actin cytoskeleton. Lastly, we measured the secretion of S100B induced by DMSO in the presence of an inhibitor of neutral sphingomyelinase 2, a key enzyme for brain exosome formation [53]. Together, these data point to the possibility that the secretion of S100B occurs by an exosomal pathway. The putative vesicular exportation of S100B is indicated in Figure 9. However, the exocytic way of S100B secretion from astrocytes deserves further and detailed investigation with appropriate vesicle markers, as carried out in serum exosomes containing the S100B protein [27,54]. In addition, it is necessary to consider the possibility of more than one secretion process. Cytokines, for example, can be released in exosomes or not, depending on cell preparation and stimulus [55]. 

Although DMSO and forskolin induced a similar profile of Ca^2+^ response, forskolin but not DMSO caused a dramatic change in astrocyte morphology, as observed by phase-contrast microscopy or F-actin staining. It is important to emphasize that the effect of forskolin on the cytoskeleton is reversed by lysophosphatidic acid, which, per se, also causes an increase in S100B secretion [7]. Once more, these data suggest that the S100B secretion profile in astrocytes is not necessarily associated with obvious changes in cell morphology, at least in primary astrocyte cultures. 

Finally, we investigated extracellular levels of S100B in the cerebrospinal fluid of rats after intracerebroventricular infusion of DMSO and in acute hippocampal slices exposed to DMSO. We found that 1% DMSO increased CSF S100B content without significantly affecting the serum S100B content, reinforcing the idea that changes in CSF S100B content are not necessarily accompanied by serum variations in this protein. Moreover, acute hippocampal slices incubated with 1% DMSO secreted more S100B, as did cultured astrocytes. These findings are interesting because DMSO, at different concentrations, is commonly used as a vehicle (and control) in ICV drug administration and in vitro assays. Cell-specific Ca^2+^ responses were reported in acute hippocampal slices [56], as was S100B secretion [20]. The S100B-secreting property of DMSO should be added to the list of potential therapeutic applications of this compound, particularly considering the neuroprotective effects of S100B in traumatic brain damage [57]. Moreover, DMSO has been recently proposed to modulate NMDA receptors in an Alzheimer’s disease model [21].

## 4. Materials and Methods

Material: Poly-L-lysine, monoclonal anti-S100B antibody (SH-B1), propidium iodide, methyl thiazolyl diphenyl-tetrazolium bromide (MTT), ethylene glycol-bis (2-aminoethyl ether)-N,N,N′,N′-tetraacetic acid (EGTA), ionomycin, A23187, BAPTA-AM, thapsigargin, forskolin, GW4869, verapamil, cobalt chloride, rhodamine-phalloidin, fura-2-AM, and pluronic F-127 were purchased from Sigma (St. Louis, MO, USA). Fetal calf serum (FCS), Dulbecco’s modified Eagle’s medium (DMEM), and other materials for cell culture were purchased from Gibco (Carlsbad, CA, USA). Polyclonal anti-S100B and anti-rabbit peroxidase-conjugated antibodies were purchased from DAKO and GE, respectively. DMSO was purchased from Merck (Darmstadt, Germany).

Dilution of the pharmacological modulators: All of the insoluble compounds were diluted in a final concentration of less than 0.1% DMSO.

Cell culture: Primary astrocyte cultures from Wistar rats were prepared as previously described [23]. Procedures were in accordance with the NIH Guide for the Care and Use of Laboratory Animals and were approved by the local authorities. Briefly, the cerebral cortex of newborn (1–2 days old) Wistar rats were removed and mechanically dissociated in Ca^2+^- and Mg^2+^-free balanced salt solution, pH 7.4, containing (in mM) 137 NaCl; 5.36 KCl; 0.27 Na_2_HPO_4_; 1.1 KH_2_PO_4_; and 6.1 glucose. The cortices were cleaned of meninges and mechanically dissociated by sequential passage through a Pasteur pipette. After centrifugation at 1000 rpm for 5 min, the pellet was resuspended in DMEM (pH 7.6) supplemented with 8.39 mM HEPES, 23.8 mM NaHCO3, 0.1% amphotericin, 0.032% gentamicin, and 10% FCS. Cultures were maintained in vitro at 37 °C in DMEM containing 10% FCS in 5% CO_2_/95% air and were allowed to grow to confluence (15 days in vitro).

Culture of cells on coverslips for calcium measurements: Confluent primary cortical astrocyte cultures were trypsinized (0.25% trypsin/EDTA), replated onto 25 mm poly-L-lysine-coated coverslips, and employed in experiments within two days. 

Calcium measurements by real-time fluorescence microscopy: For intracellular calcium measurements, cells were incubated with appropriate indicator in microscopy buffer containing (in mM) 1.5 CaCl_2_, 130 NaCl, 5.6 KCl, 0.8 MgSO_4_, 1 Na_2_HPO_4_, 25 glucose, 2 HEPES, and 2.5 NaHCO_3_, pH 7.3. For cytoplasmic calcium measurements, cells were loaded with Fura 2-AM (5 µM) in microscopy buffer containing 5 µL nonionic surfactant Pluronic F-127 (0.02%) for 30 min at room temperature. After incubation, cells were rinsed and placed in a Leiden coverslip chamber on the microscope stage. The temperature of the specimen was maintained at 37 °C during the experiments. Calcium levels were investigated in isolated cells using an inverted microscope (TE300 Nikon, Osaka, Japan) coupled to a high-resolution cooled CCD camera (Roper Sci or CoolSnap, both from Princeton Instruments, Thousand Oaks, CA, USA) and controlled by imaging software BioIP 1.0, (Wilmington, DE, USA). Cells were stimulated with either 0.5% DMSO, 2 mM EGTA, or 10 μM forskolin. Fura 2-AM (Fura 2) is a calcium-specific ratio metric fluorescent dye. The fluorophore was excited continuously at two wavelengths, 340 and 380 nm. When Fura-2 is bound to Ca^2+^, the fluorescence at 340 nm increases and the fluorescence at 380 nm decreases. Thus, the 340/380 nm ratio corresponds to variations in cytosolic calcium. Calibrations were performed using digitonin (100 µM) to obtain maximum fluorescence (Rmax) and MnCl_2_ (2 mM) for minimum fluorescence (Rmin) of the system.

Surgical procedure for intracerebroventricular injection (ICV) DMSO infusion: Procedures were in accordance with the NIH Guide for the Care and Use of Laboratory Animals and were approved by the local authorities. Adult (60 days old) Wistar rats were used. For ventricle access, the animals were anesthetized with ketamine/xylazine (75 and 10 mg/Kg, respectively, i.p.) and placed in a stereotaxic apparatus. A midline sagittal incision was made in the scalp, and one burr hole was drilled in the skull over the right lateral ventricle. The following coordinates were used: 0.9 mm posterior to bregma, 1.5 mm lateral to the sagittal suture, 3.6 mm beneath the brain’s surface. Rats received 2 µL ICV of pure DMSO or phosphate-buffered saline (control). Assuming a CSF volume of 200 µL (excluding subarachnoid space), the final DMSO concentration would be approximately 1%. After the surgical procedure, rats were kept in a stereotactic holder for 1 h, and the CSF was obtained by puncture of the cisterna magna using an insulin syringe. A maximum volume of 30 μL was collected over a 3 min period to minimize the risk of brain stem damage. Cerebrospinal fluid samples were frozen (−20 °C) until S100B analysis. Blood samples were obtained with an intracardiac puncture, and the animals were killed by decapitation. The blood samples were incubated at room temperature (25 °C) for 5 min and centrifuged at 3200 rpm for 5 min. The serum was stored at −20 °C until the day of analysis.

Hippocampal slices: Hippocampal slices were prepared as previously described [20]. Sixty-day-old Wistar rats were killed by decapitation, and their brains were removed and placed in cold HEPES-buffered saline medium with the following composition (in mM): 120 NaCl; 2 KCl; 1 CaCl_2_; 1 MgSO_4_; 25 HEPES; 1 KH_2_PO_4_; and 10 glucose, adjusted to pH 7.4, and previously aerated with O_2_. The hippocampi were dissected, and transverse slices of 0.3 mm were obtained using a McIlwain Tissue Chopper. Slices were transferred immediately to 24-well culture plates containing 0.3 mL of saline and only one slice. The medium was changed with fresh saline medium every 15 min at room temperature (25 °C). Following a 120 min equilibration period, the medium was removed and replaced with HEPES-buffered medium plus or minus DMSO for 60 min at 30 °C in a warm plate. Subsequently, 10 μL of medium was collected and stored at −20 °C until S100B measurement.

S100B measurement: S100B was measured by ELISA, as previously described [58]. Briefly, 50 μL of the sample plus 50 μL of Tris buffer were incubated for 2 h on a microtiter plate coated once with monoclonal anti-S100B. Polyclonal anti-S100 was incubated for 30 min, and then peroxidase-conjugated anti-rabbit antibody was added for 30 min. The color reaction with OPD was measured at 492 nm. The standard S100B curve ranged from 0.002 to 1 ng/mL.

Astrocyte morphology: After 1 h of drug treatment, the cells were fixed for 20 min with 4% paraformaldehyde in phosphate buffer (PBS), rinsed with PBS, and permeabilized for 10 min in PBS containing 0.2% Triton X-100. Fixed cells were then blocked for 60 min with PBS containing 5% bovine serum albumin and incubated for 20 min with rhodamine-phalloidin at 2.5 U/mL. Cells were viewed with a Nikon inverted microscope, and images were transferred to a computer with a digital camera (Sound Vision Inc., Wayland, MA, USA). All images are representative fields from four experiments carried out in triplicate.

MTT reduction assay: Cells were treated with 50 μg/mL methylthiazolyldiphenyl-tetrazolium bromide (MTT) for 30 min in 5% CO_2_/95% air at 37 °C. The medium was then removed, and MTT crystals were dissolved in DMSO. Absorbance values were measured at 560 and 650 nm. The reduction in MTT was calculated as (abs 560 nm)–(abs 650 nm).

Propidium iodide uptake assay: Cells were treated with DMSO plus 7.5 μM propidium iodide and incubated for 6 h. The cells were viewed with a Nikon inverted microscope with a TE-FM Epi-Fluorescence accessory, and images were transferred to a computer with a digital camera. All images are representative fields from at least three experiments carried out in triplicate. Optical density was determined with Optiquant version 02.00 software (Packard Instrument Company, Palo Alto, CA, USA). Density values obtained were expressed as density light units.

Data analysis of propidium iodide uptake assay, astrocyte morphology, and calcium measurements by real-time fluorescence microscopy: We used a double-blind design at all stages to perform and analyze these assays. The experiments were conducted by a different researcher than the one whoacquired the images and analyzed the data. Only the researchers who conducted the experiments knew which sample belonged to each experimental group.

Statistical analysis: Parametric data are reported as mean ± standard error and were analyzed by Student’s *t*-test (when two groups were considered) or by one-way analysis of variance (ANOVA) in SPSS-16.0. Data from S100B measurements were log-transformed to satisfy the assumption of statistical tests when necessary. Tests are specified in the legends, assuming *p* < 0.05. 

## 5. Conclusions

Our data show and consolidate the concept that astroglial S100B secretion (herein induced by forskolin or DMSO) is triggered by increases in intracellular Ca^2+^ and, in addition, point out that this increase is due to Ca^2+^ mobilization from the endoplasmic reticulum. In fact, like forskolin, DMSO caused a biphasic response of Ca^2+^ mobilization based on Fura-2 fluorescence assay. The DMSO-induced S100B secretion was confirmed Ahippocampal slices. Moreover, inhibition of plasma membrane Ca^2+^ channels, involved in the Ca^2+^ replenishment of internal sores, resulted in decreased S100B secretion. Our data support a nonclassical vesicular export of S100B by exocytosis. Our work might contribute to the understanding of the mechanism of secretion of S100B, a protein widely used as a marker of astroglial activation in experimental models and clinical studies of brain disorders.

## Data Availability

The data that support the findings of this study are available from the corresponding author, M.C.L., upon reasonable request.

## Supplementary Materials

The following supporting information can be downloaded at: https://www.mdpi.com/article/10.3390/ijms242316576/s1.
