# Supplementary material for: Astroglial S100B Secretion Is Mediated by Ca2+ Mobilization from Endoplasmic Reticulum: A Study Using Forskolin and DMSO as Secretagogues
Source: Int J Mol Sci. 2023 Nov 21;24(23):16576. doi: 10.3390/ijms242316576 (PMC10706453; doi:10.3390/ijms242316576)
Supplement: Supplementary file 1 [file ijms-24-16576-s001.zip › ijms-2641670-supplementary.pdf]

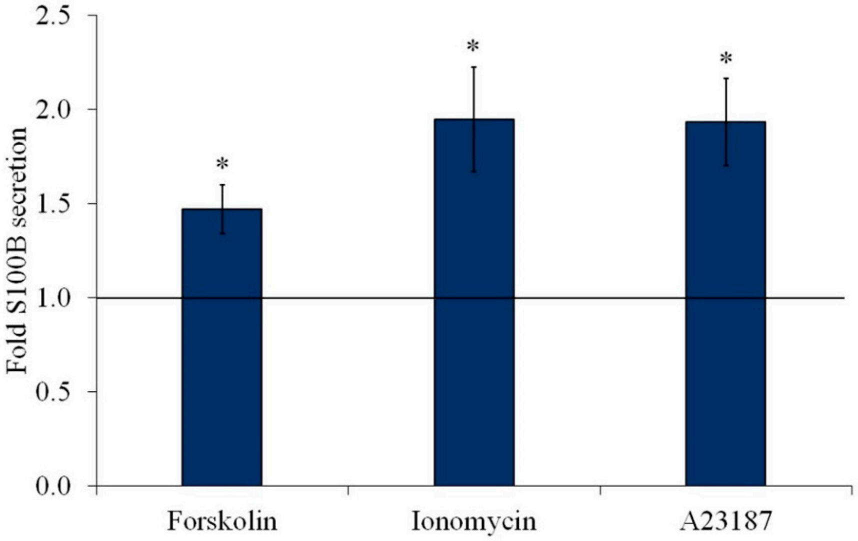

Figure S1: S100B is stimulated by forskolin and Ca^{2+} ionophores in 15 min of exposure. Rat cortical astrocytes were cultured in DMEM containing 10% FCS. The medium was replaced by DMEM without serum in the presence or absence of 10 μM forskolin, 1 μM ionomycin, or 1 μM A23187. S100B was measured by ELISA at 1 h. * Significantly different from basal secretion (Student t-test followed by Bonferroni's adjustment, $p < 0.05$). For all these results, each value represents the mean (± standard error) of at least 5 independent experi-ments performed in triplicate.

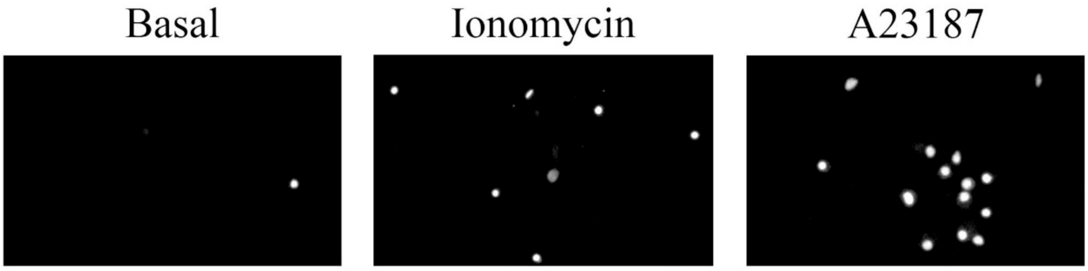

Figure S2: Ca^{2+} ionophores compromise cell integrity in 6 h of exposure. Rat cortical astrocytes were cultured in DMEM containing 10% FCS. Representative propidium iodide fluorescent images of astrocytes after 6 h of incubation in serum-free DMEM with 1 μM ionomycin or 1 μM A23187 (10X magnification).

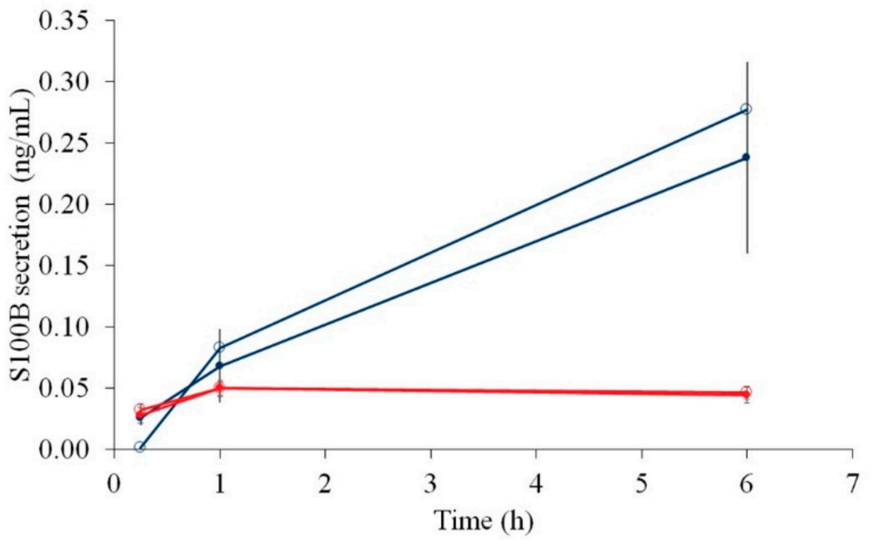

Figure S3: Ethanol does not induce S100B secretion in the presence or absence of serum. Rat cortical astrocytes were cultured in DMEM containing 10% FCS. After confluence, the medium was replaced by DMEM without serum (blue) or DMEM/10% FCS (red). Basal (solid circles) or ethanol 1% (open circles) S100B secretion was measured by ELISA at 15 min, 1 h, and 6 h. Statistical analysis was carried out by repeated measures of ANOVA.

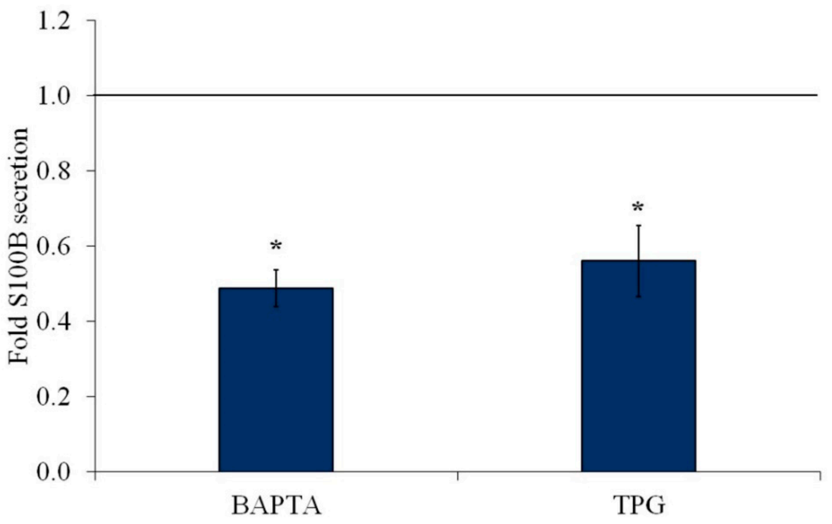

Figure S4: S100B secretion induced by serum deprivation is reduced by intracellular Ca^{2+} modulators. Rat cortical astrocytes were cultured in DMEM containing 10% FCS. After confluence, 10 µM BAPTA-AM or 1 µM thapsigargin were incubated for 15 min before media replacement. Media were then replaced by DMEM without serum containing the drug previously incubated. S100B was measured by ELISA after 1 h. Basal S100B secretion was assumed as 1 in each experiment. * Significantly different from serum-deprivation S100B secretion (one-way ANOVA followed by Dunnett's test, assuming $p < 0.05$). Each value represents the mean (± standard error) of at least 5 independent experiments performed in triplicate.

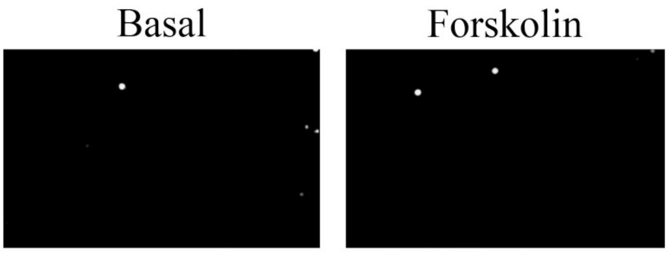

Figure S5: Forskolin do not compromise cell integrity in 6 h of exposure. Rat cortical astrocytes were cultured in DMEM containing 10% FCS. Representative propidium iodide fluorescent images of astrocytes after 6 h of incubation in serum-free DMEM with 10 μM forskolin (10X magnification).

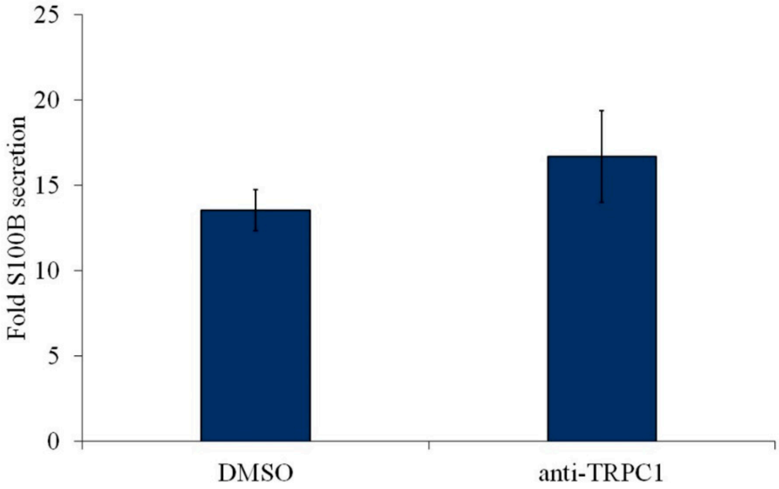

Figure S6: DMSO-induced S100B is not blocked by anti-TRPC1. Rat cortical astrocytes were cultured in DMEM containing 10% FCS. Cells were treated with anti-TRPC1 for 15 min before media replacement. Media were then replaced by DMEM without serum containing 0.5% DMSO and anti-TRPC1. S100B was measured by ELISA after 1 h. Basal S100B secretion was assumed as 1 in each experiment. Statistical analysis was made by one-way ANOVA. Each value represents the mean (± standard error) of at least 5 independent experiments performed in triplicate.

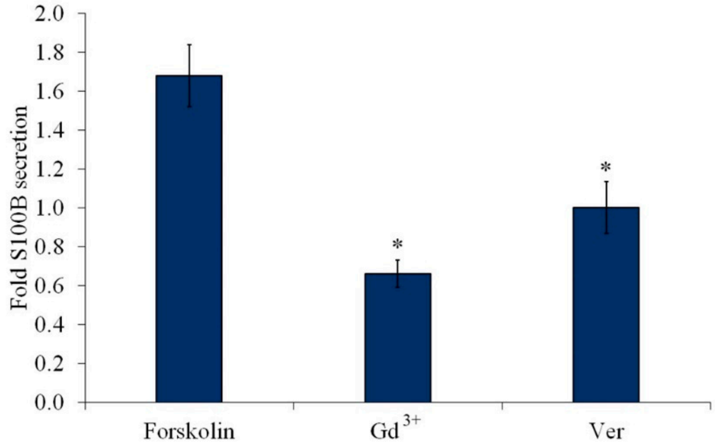

Figure S7: Forskolin-induced S100B secretion is affected by Ca^{2+} channel blockers of plasma membrane. Rat cortical astrocytes were cultured in DMEM containing 10% FCS. After confluence, the medium was replaced by the DMEM without serum, and forskolin was added at a final concentration of 10 μM. Fold S100B secretion induced by forskolin (assuming basal S100B secretion as 1 in each experiment) in the presence of channel blockers: 0.5 mM, $GdCl_3$ (Gd^{3+}) or 50 μM verapamil (Ver). Each value is the mean (± standard error) of at least 5 independent experiments performed in triplicate. * Significantly different from forskolin-induced secretion (one-way ANOVA followed by Dunnett's test, assuming $p < 0.05$).
